# EVERYDAY DISCRIMINATION AND KIDNEY FUNCTION AMONG OLDER ADULTS: EVIDENCE FROM THE HEALTH AND RETIREMENT STUDY

**DOI:** 10.1093/geroni/igz038.690

**Published:** 2019-11-08

**Authors:** Ryon J Cobb, Roland J Thorpe, Keith C Norris

**Affiliations:** 1 University of Texas at Arlington, Arlington, Texas, United States; 2 The John Hopkins University, Baltimore, Maryland, United States; 3 UCLA School of Medicine, Los angeles, California, United States

## Abstract

Background: The current study examines the cross-sectional association between everyday discrimination and kidney function among older adults. Methods: We use cross-sectional data from a nationally representative sample of older adults to examine this relationship. Our measure of kidney function derives from the estimated glomerular filtration rate (eGFR) obtained by the Chronic Kidney Disease Epidemiology Collaboration equation, while our indicator of everyday discrimination is drawn from self-reports. Results: Results from our ordinary least squared regression models reveals that, after adjusting for demographic characteristics, everyday discrimination was associated with lower mean eGFR (β=-.79; S.E.: .34). The relationship between everyday discrimination and kidney function was not explained by cardiovascular, metabolic, or economic factors. Conclusions: Findings suggest this study suggest that everyday discrimination may be a unique risk factor for poorer kidney function among older adults. Because these findings are cross-sectional, additional research is needed to determine whether the observed associations persist over time.

